# Hints on ATGL implications in cancer: beyond bioenergetic clues

**DOI:** 10.1038/s41419-018-0345-z

**Published:** 2018-02-22

**Authors:** Rolando Vegliante, Luca Di Leo, Fabio Ciccarone, Maria Rosa Ciriolo

**Affiliations:** 1 Department of Biology, University of Rome “Tor Vergata”, Via della Ricerca Scientifica, 00133 Rome, Italy; 2 IRCCS San Raffaele ‘La Pisana’, Rome, Italy

## Abstract

Among metabolic rearrangements occurring in cancer cells, lipid metabolism alteration has become a hallmark, aimed at sustaining accelerated proliferation. In particular, fatty acids (FAs) are dramatically required by cancer cells as signalling molecules and membrane building blocks, beyond bioenergetics. Along with *de novo* biosynthesis, free FAs derive from dietary sources or from intracellular lipid droplets, which represent the storage of triacylglycerols (TAGs). Adipose triglyceride lipase (ATGL) is the rate-limiting enzyme of lipolysis, catalysing the first step of intracellular TAGs hydrolysis in several tissues. However, the roles of ATGL in cancer are still neglected though a putative tumour suppressor function of ATGL has been envisaged, as its expression is frequently reduced in different human cancers (*e.g.*, lung, muscle, and pancreas). In this review, we will introduce lipid metabolism focusing on ATGL functions and regulation in normal cell physiology providing also speculative perspectives on potential non-energetic functions of ATGL in cancer. In particular, we will discuss how ATGL is implicated, mainly through the peroxisome proliferator-activated receptor-α (PPAR-α) signalling, in inflammation, redox homoeostasis and autophagy, which are well-known processes deregulated during cancer formation and/or progression.

## Facts

- ATGL is the rate-limiting enzyme of lipolysis, mainly expressed in adipose tissues but virtually functioning in all other organs.
- ATGL has been found downregulated in several human cancers including lung and pancreas.
- In most immune cells ATGL activity contributes to pro-inflammatory responses mainly providing precursors for eicosanoids. In non-immune cells (*e.g.*, myocytes, hepatocytes and adipocytes) the ATGL-PPAR-α axis maintains an anti-inflammatory phenotype.
- ATGL favours redox homoeostasis in many cell types mainly through a PPAR-α-mediated control of antioxidant enzymes.
- ATGL triggers autophagy through SIRT1 in hepatocytes and its involvement in lipophagy facilitates lipid droplets mobilisation.

## Open questions

- Identification of upstream signalling pathways that account for ATGL downregulation in cancer
- Analysis of the ATGL-PPAR-α axis in cancer development and/or progression
- Identification of downstream molecular targets linking ATGL downregulation to tumorigenesis

## Introduction

Lipids, including triacylglycerols (TAGs) and cholesterol, essentially derive from dietary sources and are transported in the blood mainly packaged in lipoprotein complexes. Free fatty acids (FAs) are obtained from TAGs-rich lipoprotein through the action of lipoprotein lipases, located at the capillary walls of most tissues, or by lysosomal acidic hydrolysis following lipoprotein endocytosis. Moreover, FAs also derive from *de novo* biosynthesis and those released by the adipose tissue are substrates for mitochondrial β-oxidation in most cell types. Beyond energetic purposes, FAs are essential for membranes biosynthesis and also serve as signalling molecules^1^.

**Fig. 1:**
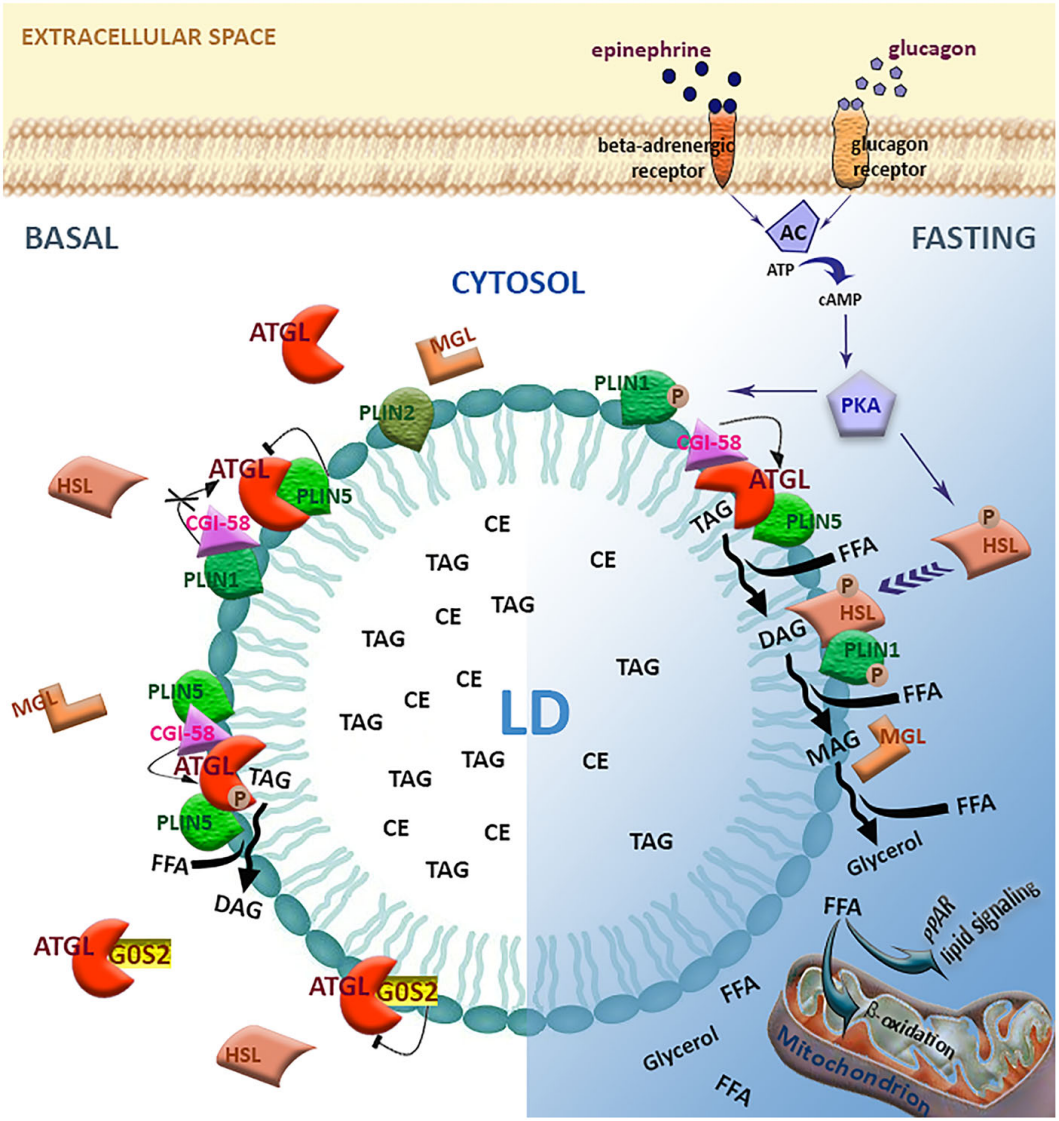
Canonical lipolytic function of ATGL in adipocytes. Schematic representation of lipid droplet (LD) components and the lipolytic machinery. LDs are organelles storing triacylglycerols (TAGs) and cholesteryl esters (CEs) and are composed of a phospholipid monolayer anchoring different proteins, including the perilipins (PLINs). In the unstimulated condition, ATGL is inhibited by the association with PLIN5 and G0S2. However, a basal TAG hydrolysis by ATGL could be achieved through AMPK-mediated phosphorylation and/or interaction with CGI-58, when the latter is not associated with PLIN1. In the fasted state, the hormones epinephrine or glucagon bind to their cognate receptors stimulating the adenylyl cyclase (AC) activity. Increased cAMP levels activate cAMP-dependent protein kinase (PKA), which phosphorylates ATGL, PLIN1 and hormone-sensitive lipase (HSL). Following PLIN1 phosphorylation, CGI-58 is released and thus it can further activate ATGL. PKA-mediated phosphorylation of HSL favours its association with LDs and its activity, leading to diacylglycerol (DAG) conversion into monoacylglycerol (MAG). The lipolytic cascade culminates with the MAG lipase (MAGL) activity, which induces the release of free fatty acid (FFA) and glycerol. FFAs are then delivered to mitochondria to produce energy through β-oxidation or used as signalling molecules for nuclear receptors, such as peroxisome proliferator-activated receptors (PPARs)

Intracellular fat depots consist of lipid droplets (LDs), which store neutral lipids such as TAGs and cholesteryl esters to avoid lipotoxicity. LDs are dynamic organelles with complex biogenesis and multiple functions, present in many cell types beyond adipocytes^2–4^. Consistently, the enzymatic machinery for LDs remodelling/utilisation to release FAs is ubiquitously expressed and equipped with specific hydrolases designated lipases. The adipose triglyceride lipase (ATGL) selectively catalyses the first and rate-limiting step of intracellular TAGs hydrolysis to generate diacylglycerol (DAG) and FAs^5,6^. DAGs are then sequentially hydrolysed by the hormone-sensitive lipase (HSL) and the monoacylglycerol lipase (MAGL), achieving the release of FAs and glycerol. Overall, this process is known as lipolysis (Fig. 1)^7,8^. The molecular mechanisms involved in lipolysis are well-characterised in adipocytes. Indeed, fasting and hormone stimulation, including epinephrine and glucagon, trigger the activation of cAMP-dependent protein kinase A (PKA). The phosphorylation of HSL by PKA is required for translocation to LDs and consequent activation^9^. On the contrary, ATGL hydrolytic activity on LDs occurs also in a hormone-independent fashion and is further enhanced upon fasting, depending on AMPK-mediated phosphorylation^10^, and following β-adrenergic stimulation via PKA-mediated phosphorylation (Fig. 1)^11^.

ATGL is a member of the patatin-like phospholipase domain containing (PNPLA) family, specifically codified by the *PNPLA2* gene and highly expressed in adipose tissue, whereas moderate to low levels are detectable in all other tissues, including liver, heart and skeletal muscle^5^. The relevance of ATGL for whole-body energetics is confirmed by TAGs systemic accumulation in ATGL knockout (KO) mice, which face premature death from cardiac dysfunction due to massive fat accumulation^12^. Organ-specific features of ATGL KO mice include hepatic steatosis^13^ and altered insulin signalling pathway in adipose tissue, liver and skeletal muscle^14,15^. In humans, *PNPLA2* gene bi-allelic loss-of-function mutations also cause extensive LDs accumulation in several organs leading to a disorder named “neutral lipid storage disease with myopathy” (NLSDM), characterised by progressive myopathy, cardiomyopathy and hepatomegaly^16,17^.

Most aspects of ATGL functions in lipolysis have been characterised in adipocytes. ATGL association with LDs in basal conditions is primarily mediated by a highly conserved C-terminal hydrophobic sequence^5,18^. Moreover, an intricate crosstalk with different protein partners controls ATGL localisation and activity. Indeed, the interaction with comparative gene identification-58 (CGI-58) is known to stimulate ATGL activity^19^ while the small basic protein G0/G1 switch gene 2 (G0S2) was identified as a selective inhibitor of ATGL impeding substrate accessibility^19,20^. Furthermore, the LD-associated scaffold proteins perilipins (PLINs) are implicated in the regulation of ATGL function. PLIN1 is highly expressed in adipose tissue and, in basal conditions, sequesters CGI-58, which is released by hormone stimulation with consequent activation of ATGL^21,22^. PLIN5 is instead expressed in muscle, liver and brown adipose tissue and, although promoting ATGL association with LDs, impairs its hydrolase activity^23^.

ATGL-mediated lipolysis releases FAs that are used for β-oxidation as well as intracellular signalling. Indeed, FAs are cognate ligands for nuclear receptors/transcription factors, among which the peroxisome proliferator-activated receptor (PPAR) family is the most characterised^24^. In particular, ATGL activity is associated with increased levels of PPAR-α downstream targets, which are mainly involved in increased uptake and oxidation of lipids^25^. Consistently, ATGL-released free FAs favour energy expenditure through the upregulation of mitochondrial biogenesis and β-oxidation genes in combination with peroxisome proliferator-activated receptor gamma co-activator 1-alpha (PGC-1α) in cardiac muscle, liver and adipose tissue^25–27^. An alternative mechanism describes ATGL-mediated activation of Sirtuin 1 (SIRT1) as the linking event that triggers PGC-1α/PPAR-α signalling^28^. Mitochondrial functionality in brown adipocytes and pancreatic β cells is instead regulated by ATGL-mediated activation of PPAR-δ^27,29^. Along with the described ATGL/PPAR-α signalling impinging on mitochondrial metabolism, this axis impacts on additional processes that will be deeply examined throughout this review.

## Metabolic rewiring and ATGL deregulation in cancer

Metabolic reprogramming is a key feature of cancer cells to sustain fast proliferation rate, which is at the basis of tumour progression^30,31^. In order to cope with the required energetic demand, most cancer cells exploit a faster ATP production by enhancing the glycolytic rate rather than the oxidative phosphorylation. This metabolic adaption is defined as Warburg effect, or aerobic glycolysis^32^. Importantly, the metabolic requirement of cancer is not uniquely limited to energetics as it also accounts for the generation of new cells in the tumour mass by microenvironment rearrangement and augmented macromolecule synthesis^31^. In this regard, glutamine is largely engaged as nitrogen donor for nucleotide and amino-acid biosynthesis while sustaining mitochondrial metabolism^33^. Interestingly, also increased *de novo* lipid synthesis has been established as key metabolic footprint of nearly all cancers and is achieved through the upregulation of lipogenic enzymes. Unlike most adult tissues except adipose tissue and liver, cancer cells reactivate FAs synthesis for the generation of membranes building blocks and for the sustenance of oncogenic lipid signalling and protein post-translational modification^34^. Remarkably, also lipid catabolic pathways are re-adapted in transformed cells. For instance, non-glycolytic tumours, like prostate cancer and diffuse large B-cell lymphoma, are highly dependent on mitochondrial β-oxidation^35,36^. Moreover, elevated activity of lipases, including phospholipase A2 (producing lysophospholipids and FAs from glycerophospholipids) and phospholipase D (producing phosphatidic acid and free choline from phosphatidylcholine), has been documented^37,38^.

Although the fundamental role of ATGL in LDs catabolism has been extensively investigated in the last decade, only recent evidence has highlighted a deregulation of ATGL in cancer specimens. However, the few data available on the mechanisms by which ATGL might impinge on cancer formation and progression are still elusive and controversial.

Most of *in vitro* studies have proposed pro-neoplastic features of ATGL. Consistently, reduced proliferation and invasiveness were observed upon ATGL depletion in colorectal cancer cells^39^ and non-small-cell lung carcinoma cell lines^40^. The role of ATGL in prostate cancer cells is instead ambiguous as opposite evidence has been reported up to now^41,42^. Interestingly, ATGL upregulation in breast cancer was associated with a tumour microenvironment enriched in adipocytes, contributing to aggressiveness of high-grade tumours^43^. Similarly, pancreatic ductal adenocarcinoma with elevated levels of ATGL were characterised by higher adiposity and stromal proliferation (desmoplasia)^44^.

This scenario is further complicated by the contribution of ATGL interactors to cancer. In this regard, an antitumor property for the ATGL co-activator CGI-58 was proposed. Indeed, deregulation of CGI-58 in prostate and colorectal cancer cells did not mimic the effects of ATGL on cell proliferation and invasion, suggesting that CGI-58 regulates such processes independently of ATGL^39,41,45^. On the contrary, the tumour suppressor function of G0S2 actually occurs by inhibition of ATGL, attenuating cell growth and motility in cancer cells^40^. Nevertheless, ATGL is not required for oncogene-mediated transformation of G0S2 null fibroblasts^45^.

Strikingly, recent *in vivo* insights unambiguously shed light on anti-neoplastic effects of ATGL in mouse models and human cancer. Indeed, mice lacking ATGL displayed spontaneous development of pulmonary neoplasia^46^, whereas adipose-specific ablation of both ATGL and HSL-induced liposarcoma in brown adipose tissue between 11 and 14 months of age^47^. Consistently, the expression of ATGL was found extremely reduced in human specimens of lung adenocarcinoma and lung squamous cell carcinoma with respect to normal epithelium^46^. Analogously, loss of ATGL was also depicted in pancreatic adenocarcinoma and pancreatic intraepithelial neoplasia, being progressive and stage-dependent^46^. Reduction of ATGL levels were also disclosed in malignant smooth muscle tumour (leiomyosarcoma), with respect to normal counterpart^46^. Accordingly, unpublished data from our laboratory highlighted reduced transcript levels of ATGL in both human biopsies and in a murine model of induced hepatocellular carcinoma. Although no causative molecular mechanisms for ATGL downregulation has been revealed yet, it is noteworthy to mention that the 11p15.5 chromosome arm harbouring the human *PNPLA2* gene results frequently deleted in cancer and considered a hot-spot of cytogenetic alterations in human malignancies^48,49^. Indeed, *PNPLA2* is deleted in the 38% of lung cancer and in other cancer types, including ovarian serous cystadenocarcinoma, glioblastoma, oesophageal and stomach carcinomas and paraganglioma^46^.

While the role of ATGL in cancer is still debated due to incongruence between *in vitro* and *in vivo* evidence, the oncogenic role of MAGL, the third lipolytic enzyme, in cancer is well established^50–52^. Indeed, MAGL is highly expressed in many aggressive human cancers where it orchestrates lipid signalling-mediated tumorigenesis, migration and invasion, acting as a critical regulator of metastatization^52–54^. Given this evidence, it is enticing to hypothesise that each lipase exerts functions beyond metabolism (*i.e.*, lipolysis) that account for their contribution to cancer. In this context, it is noteworthy that ATGL affects many cellular processes including inflammation, oxidative stress and autophagy, which are highly perturbed in malignant cells. These ATGL effects highly rely on the activation of PPAR-α signalling, are largely conserved among tissues and may correlate with its contribution to tumour biology, as reported in the following sections.

## ATGL and inflammation

Inflammation is critically involved in cancer initiation and progression. In fact, sites of infection or chronic injury can originate solid tumours due to local persistent inflammation, which induces genome instability, DNA damage and activation of cancer-promoting genes^55,56^. Alternatively, inflammatory cells can be channelled later in tumour sites, where they build up a signalling network that promotes disease progression. Consistently, inflammatory tumour microenvironment fosters survival and migration promoting the formation of blood and lymphatic vessels that feed and disseminate cancer cells^57,58^. Tumour microenvironment is directly orchestrated by cancer cells that have equipped their secretome with signalling inflammatory molecules, such as cytokines and chemokines, enabling the recruitment of immune and endothelial cells in order to further amplify inflammatory and angiogenetic processes^59^.

**Fig. 2:**
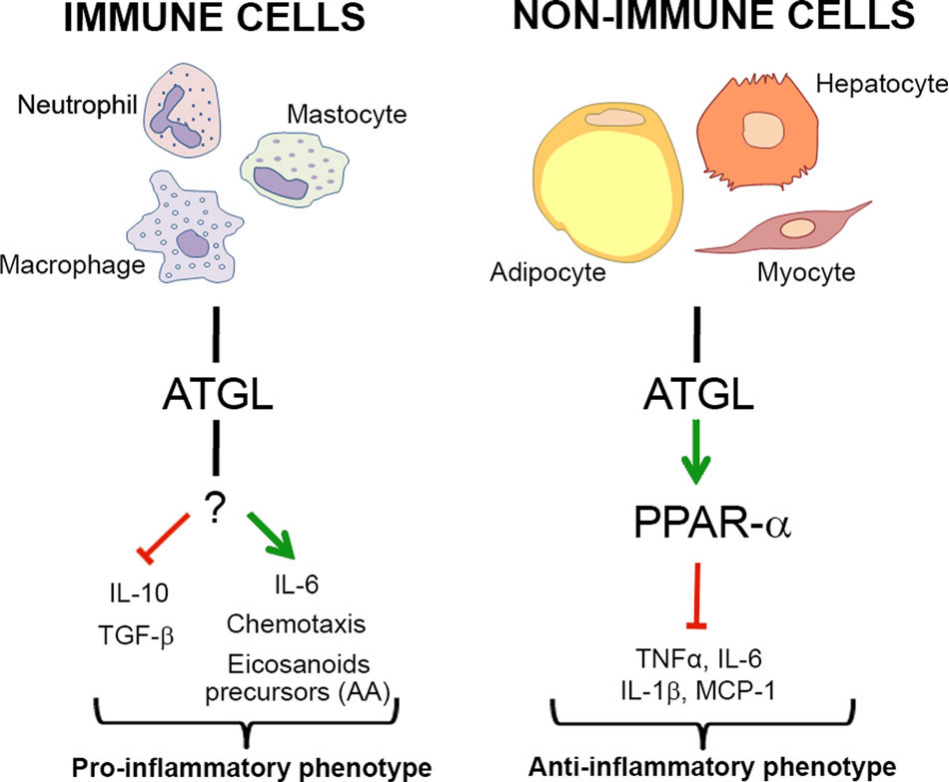
ATGL effects in inflammation. In most immune cells ATGL activity supports pro-inflammatory responses and chemotaxis mainly contributing to the production of the pro-inflammatory cytokines IL-6, and of arachidonic acid (AA), the precursor of eicosanoids. The protein effectors downstream ATGL activity are still poorly characterised in immune cells. In some non-immune cells, namely adipocytes, myocytes and hepatocytes, the ATGL-PPAR-α axis hinders the production of pro-inflammatory cytokines including IL-6, TNFα, IL-1β and MCP-1

A possible anti-neoplastic feature of ATGL can be envisaged in its ability to restrain inflammatory response in non-immune cells. In fact, heart, adipose tissue and skeletal muscle from ATGL KO mice exhibited increased basal mRNA levels of pro-inflammatory genes including tumour necrosis factor (TNF)α, interleukin (IL)-6, IL-1β and monocyte chemotactic protein 1 (MCP-1) (Fig. 2)^60–62^. Livers from ATGL KO mice also displayed augmented inflammatory response, in terms of high transcript levels of pro-inflammatory markers, after the induction of hepatic inflammation dependent on steatohepatitis or upon endotoxin challenge^63^.

Attenuation of inflammatory response mediated by ATGL in non-immune cells has been largely associated with the activation of PPAR-α signalling (Fig. 2). Consistently, myocytes and adipocytes depleted for ATGL showed decreased levels of PPAR-α-target cytokine genes^61,64^. Moreover, treatment of ATGL KO mice with PPAR-α agonists ameliorated the massive inflammatory response occurring after the induction of hepatic inflammation^63^. Therefore, the ATGL-mediated FAs release may partially contribute to the already established anti-inflammatory role of PPAR-α^65^, at least in non-immune cells.

It has to be mentioned that ATGL also participates to proper inflammatory response of immune cells. The first indirect evidence of this action derived from the prominent accumulation of LDs in leucocytes from subjects affected by NLSDM, a hallmark of the disease known as Jordan’s anomaly^17^. Later studies on animal models corroborated this evidence. Elevated content of LDs was observed in peripheral blood cells from ATGL KO mice, particularly neutrophils^66^, which exhibited an even more evident increase of LDs as well as of chemotaxis when challenged with pro-inflammatory stimuli. Notably, LDs can be a source of arachidonic acid, a precursor of the inflammatory signalling molecules eicosanoids, the biosynthesis of which is compromised by ATGL deletion/inhibition in neutrophils and mastocytes (Fig. 2)^66,67^.

Broad aspects of macrophage functionality are also affected by ATGL depletion. In particular, macrophages from ATGL KO mice show an anti-inflammatory phenotype characterised by inefficient phagocytic activity, impaired migration and decreased release of the pro-inflammatory cytokine IL-6, associated with an increase of the anti-inflammatory molecules IL-10 and TGF-β (Fig. 2)^68–70^. Interestingly, the ATGL-mediated regulation of all these processes in macrophages can underpin the attenuated formation of atherosclerotic lesions in a mouse model of atherosclerosis transplanted with bone marrow from ATGL KO mice^69^.

## ATGL and oxidative stress

Intracellular redox environment is fundamental for several biological functions, the most straightforward of which are the redox-dependent transduction pathways that control cell cycle progression, growth and death^71^. Intracellular redox status results from the balance between pro-oxidant molecules, such as reactive oxygen species (ROS) and antioxidants, namely enzymatic (*e.g.*, catalase, superoxide dismutase, glutathione peroxidase) and non-enzymatic (*e.g.*, vitamin E, glutathione) ones^72,73^. Oxidative stress is the detrimental condition occurring upon excessive ROS production or impairment of antioxidant response^74,75^. Notably, cancer cells possess inherent higher levels of ROS, which have been implicated in several aspects of tumour progression. Tumour-associated oxidative stress can be responsible for genetic and epigenetic mutations that affect expression of oncogenes and tumour suppressors^76,77^.

Mitochondrial activity is the major source of endogenous ROS with a well-recognised impact on proliferative and survival signalling pathways^78,79^. In addition, impingement of ROS on proliferation, angiogenesis or metastatization has been also ascribed to increased activity of NADPH oxidases^80–82^, a family of enzyme whose primary function is to catalyse the transfer of electrons from NADPH to oxygen generating superoxide anion radical (O_2_^.-^) and H_2_O_2._

**Fig. 3:**
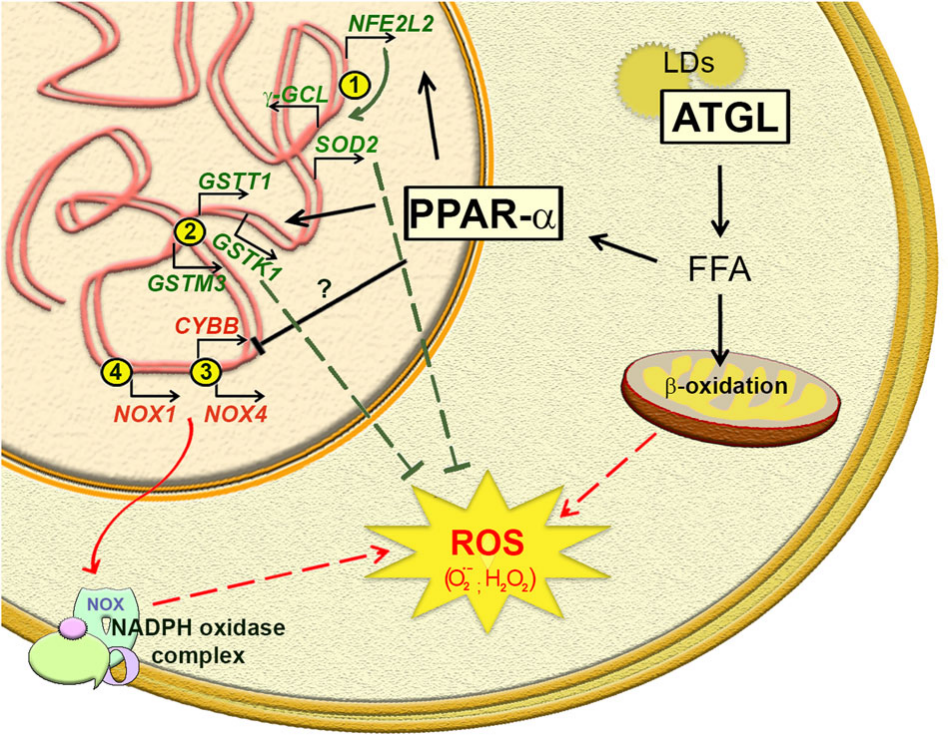
ATGL effects in redox homoeostasis. ATGL-mediated release of free fatty acids (FFAs) from lipid droplets (LDs) is a source of mitochondrial reactive oxygen species (ROS). The ATGL-PPAR-α axis mitigates oxidative stress favouring the expression of antioxidant enzymes in (1) skeletal muscle (*i.e.*,* SOD2* and *γ-GCL*, two downstream targets of NFE2L2) and in (2) intestines (*i.e.*,* GSTT1, GSTK1* and *GSTM3)*. ATGL activity impairs the expression of catalytic subunits of the NADPH oxidase complex in (3) cardiac muscle (*i.e.*,* CYBB*, codifying for NOX2 protein, and *NOX4*) and in (4) macrophages (*i.e.*,* NOX1)*. The influence of PPAR-α in these latter events has been not investigated, yet

FAs mitochondrial oxidation generates massive ATP production through oxidative phosphorylation, but how lipids influence mitochondrial redox status has not been fully elucidated yet. It can be envisaged that enhanced FAs oxidation causes sustained mitochondrial ROS emission and ATGL may have a leading role especially in cells that store large amounts of LDs. Nevertheless, this hypothesis is not supported by literature as reports mostly argue for an antioxidant role of ATGL. Heart-specific ATGL KO was associated with increased oxidative stress caused by NADPH oxidase activity^62^. Indeed, mRNA and protein levels of NOX2 and NOX4, two catalytic subunits of NADPH oxidase complexes, were raised in ATGL-deficient cardiomyocytes (Fig. 3)^62^. Similarly, increased levels of ROS were observed in ATGL-depleted macrophages as a consequence of higher expression of the NOX1 subunit (Fig. 3)^83^. Additional data highlighted the suppression of oxidative stress by the ATGL-PPAR-α pathway through the upregulation of antioxidant enzymes. ATGL KO mice displayed an increased amount of carbonylated proteins and reduced glutathione content in skeletal muscle^61^. This phenotype was due to impaired ATGL-PPAR-α signalling that caused decreased expression of PGC-1α and of nuclear factor (erythroid-derived 2)-like 2 (NFE2L2) transcription factor, whose target genes include superoxide dismutase 2 (*SOD2)* and γ-glutamylcysteine ligase (*GCLC*)^61^ (Fig. 3). In another study, intestines from ATGL KO mice showed reduced mRNA levels of PPAR-α target genes, among which are the antioxidant enzymes glutathione-S-transferases (Fig. 3)^84^. Remarkably, *PPAR-α* expression itself was recently shown to be reduced in ATGL KO mice kidney, which also displayed increased ROS levels and tubulointerstitial damage, with resulting renal dysfunction^85^.

Overall, the antioxidant function of ATGL represents a broad feature of different tissues and is mainly achieved through the activation of the PPAR-α-dependent transcriptional programmes. Antioxidant properties of PPAR-α have been already shown in different disease models. Indeed, oxidative stress following ischaemia/reperfusion injury in rat hippocampus was mitigated by PPAR-α agonist WY14643^86^, which also attenuated ROS-mediated hepatic fibrosis via catalase expression^87^. In this scenario, it is intriguing to speculate that disabling the antioxidant functions of ATGL/PPAR-α could increase ROS-dependent cancer proliferative signalling.

## ATGL and autophagy

Autophagy is a highly complex set of regulated events carried out by autophagy-related proteins (ATGs), aimed at degrading in-excess/misfolded proteins and damaged organelles in order to maintain cellular homoeostasis or to recycle macromolecules upon stress stimuli^88^. Due to its high dynamism, autophagy has a complex role in cancer, largely dependent on the stage of tumour development. Indeed, a tumour suppressor function of autophagy is linked to the clearance of damaged organelles such as mitochondria, a source of ROS leakage and DNA mutations. Conversely, by providing metabolic substrates, autophagy might have a pro-survival role in tumours facing nutrient shortage^89^.

Autophagy is also a key regulator of lipid homoeostasis: in particular, the autophagy-mediated degradation of lipids is referred to as lipophagy^90^. The first evidence of autophagy as a critical player in lipid metabolism goes back to the evidence that TAGs were increased upon autophagy impairment by *ATG5* knockdown^91^. Interestingly, LDs, which can be targets of autophagy, are surrounded by the autophagic protein microtubule-associated protein 1A/1B-light chain 3 (LC3)^92,91^. However, the role of LC3 on LDs surface is still debated because, even though it is fundamental for the autophagic/lipophagic process, LC3 is also responsible for LDs formation^93^.

Notably, ATGL *per se* seems to play an active role in lipophagy regulation. In fact, ATGL protein sequence harbours the LC3-interacting region (LIR), a motif mediating the association between autophagy receptors and LC3-coated autophagosomes^94^. ATGL/LC3 interaction contributes to the recruitment of ATGL on LDs for a more efficient LDs degradation achieved by the synergistic effect of lipolysis and lipophagy. Moreover, a recent report has demonstrated that ATGL stimulates autophagy and lipophagy via SIRT1 signalling in liver^95^. In this context, it has to be mentioned that lipid homoeostasis in liver largely relies on lipophagy and that its impairment favours hepatic steatosis, one of the leading risk factors for hepatocellular carcinoma development^91,96^. Remarkably, ATGL KO mouse models develop progressive hepatic steatosis^13^. Moreover, ATGL repression mediated by the steroid receptor RNA activator (SRA) represents the mechanism by which also this long non-coding RNA mediates hepatic steatosis^97^. These observations together with our unpublished evidence of ATGL reduction in liver cancer suggest that deregulation of ATGL lipolytic and lipophagic activity is detrimental for liver homoeostasis with potential tumorigenic consequences. Further efforts are necessary to validate this hypothesis and to extend it to other tissues that face excessive lipid accumulation, such as pancreas.

## Concluding remarks

Research work over the past decade has undoubtedly established the pivotal role of ATGL in lipid metabolism in organs other than adipose tissue. Lipid accumulation due to ATGL dysfunction affects liver and skeletal muscle causing steatohepatitis and myopathies, respectively. Moreover, diverse types of neoplasia exhibit decreased levels of ATGL or deregulated expression of its protein partners, particularly the activator CGI-58 and the inhibitor G0S2^39,40^. Most of the current *in vivo* evidence supports an anti-neoplastic role for ATGL, although the underlying up- and down-stream mechanisms have not been elucidated yet. On the contrary, other lipases, including MAGL and phospholipase D, are mainly upregulated in cancer triggering a lipid signalling network associated with enhanced tumour growth, invasion and metastasis. This inconsistency needs further clarification but the different non-energetic functions of ATGL illustrated in the present review may provide an explanation.

**Fig. 4:**
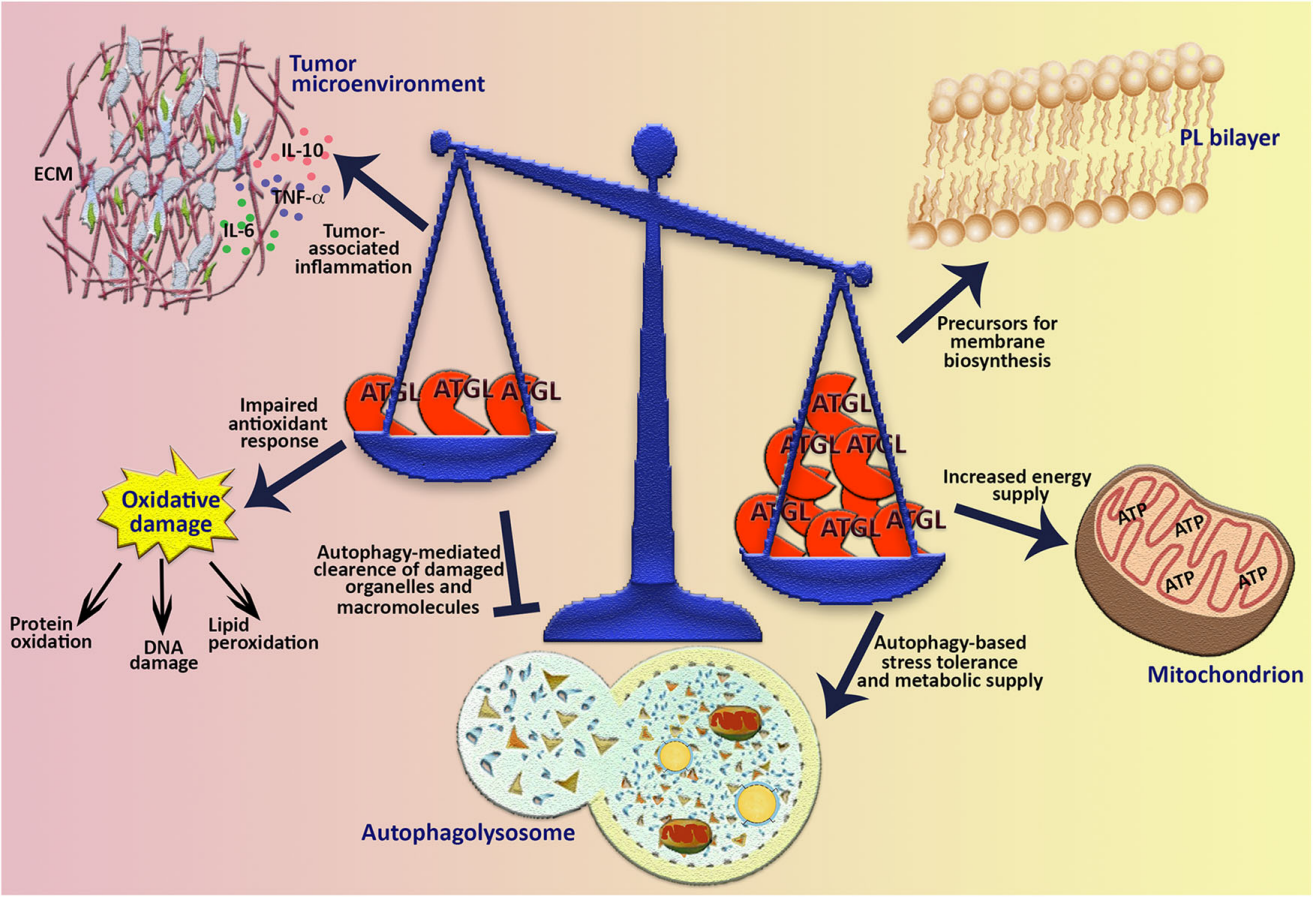
Multifaceted role of ATGL in cancer. Changes of ATGL levels can have different impact on several aspects of tumour biology. In fact, beyond providing lipids for membrane building blocks and for energy production through oxidative metabolism, ATGL could also manage tumour-associated inflammation, oxidative stress response and autophagic/lipophagic process. ECM extracellular matrix, PL phospholipids

We offered insights into several aspects of ATGL biology that are not directly dependent on energetic purposes (*i.e.*, FAs β-oxidation) and we revised all the available information about ATGL deregulation in cancer. We hypothesise that ATGL may have a broad influence on processes linked to cancer (Fig. 4), such as redox homoeostasis, inflammation and autophagy, through PPAR-α signalling. This is suggested by the evidence that the ATGL-PPAR-α axis has similar outcomes on inflammatory and antioxidant responses in several tissues, whereas tissue-specificity effects of PPAR-α target genes involved in fatty acid uptake and oxidation have been observed^25,98^. Absence of ATGL in non-immune cells is associated with oxidative stress and enhanced production of pro-inflammatory cytokines in basal and/or stimulated conditions. Considering this, the downregulation of ATGL in cancer cells might prime the set-up of an inflammatory microenvironment necessary for fibroblasts, endothelial cells and leucocytes recruitment and the induction of redox-based proliferative signalling, two events that favour cancer formation and progression^99,100^.

Although a connection between ATGL and autophagy exists, no definitive molecular mechanism has been established yet. A tentative speculation on the pro-lipophagic role of ATGL in the repression of hepatosteatosis and of liver carcinogenesis has been provided. However, no evidence on ATGL KO mice and on the actual regulation of autophagic/lipophagic flux in pathological conditions has been provided. Further investigation may be interestingly addressed to understand whether ATGL contributes to the pro-autophagic action of PPAR-α signalling. Indeed, it has been demonstrated that treatment with PPAR-α agonists increases LC3 protein levels in fed mice^101^ as well as transcript levels of autophagic genes in a murine model of acute liver failure, leading to an active autophagic flux^102^.

Overall, some tumours might take advantage from ATGL deregulation to suppress the non-energetic functions herewith described, which would otherwise hinder tumour promotion/progression. At the same time, ATGL downregulation might contribute to the switch from mitochondrial metabolism to a glycolytic phenotype typical of many cancers. Furthermore, dampening ATGL activity may be necessary for those tumours that rely on high amount of intracellular LDs. In fact, LDs accumulation in cancer has been associated with resistance to therapeutic treatments^103^ and to endoplasmic reticulum stress conditions^104^.

Although the molecular mechanisms underlying ATGL additional roles in cancer biology are still elusive, its anti-neoplastic features could be useful for targeting cancer cells. Particularly, it could be intriguing to use approaches aimed at increasing ATGL expression/activity or mimicking its functions. In this regard, the alkaloid berberine and the steroid hormone dehydroepiandrosterone (DHEA) have been documented to increase ATGL expression in adipose tissue *in vitro* and *in vivo*, respectively, and to ameliorate lipid mobilisation^105,106^. Furthermore, considering the role of PPAR-α in mediating ATGL signalling, the use of PPAR-α agonists, such as fenofibrate, could be an enticing approach to counteract cancer progression. Indeed, fenofibrate was reported to disturb glioma cell growth and melanoma metastatic activity both *in vitro* and *in vivo*^107–109^. Overall, future efforts are needed to deeply characterise ATGL molecular mechanisms in cancer biology and to develop selective therapeutic strategies.
